# Identification of miR-215 mediated targets/pathways via translational immunoprecipitation expression analysis (TrIP-chip)

**DOI:** 10.18632/oncotarget.4425

**Published:** 2015-06-10

**Authors:** Andrew Fesler, Xiao Xu, Xiao Zheng, Xiaodong Li, Jingting Jiang, James J. Russo, Jingfang Ju

**Affiliations:** ^1^ Department of Pathology, Stony Brook University, School of Medicine, Stony Brook, NY, USA; ^2^ Icahn School of Medicine at Mount Sinai, New York, NY, USA; ^3^ The Third Affiliated Hospital, Soochow University, China; ^4^ Center for Genome Technology and Biomolecular Engineering, Department of Chemical Engineering, Columbia University, New York, NY, USA

**Keywords:** miR-215, translation, protein chaperone, HSP70

## Abstract

Steady state mRNA expression profiling can identify the majority of miRNA targets. However, some translationally repressed miRNA targets are missed and thus not considered for functional validation. Therefore, analysis of mRNA translation can enhance miRNA target identification for functional studies. We have applied a unique approach to identify miRNA targets in a small number of cells. Actively translating mRNAs are associated with polyribosomes and newly synthesized peptide chains are associated with molecular chaperones such as HSP70s. Affinity capture beads were used to capture HSP70 chaperones associated with polyribosome complexes. The isolated actively translating mRNAs were used for high throughput expression profiling analysis. miR-215 is an important miRNA in colorectal cancer and loss of miR-215 is significantly associated with prognosis of this disease. miR-215 suppresses the expression of several key targets. We utilized the affinity capture approach to isolate miR-215 mediated mRNA target transcripts. This approach provides a unique way to identify targets regulated by non-coding RNAs and RNA binding proteins from a small number of cells.

## INTRODUCTION

Gene expression is precisely regulated at multiple levels. With regard to protein synthesis, post-transcriptional and translational controls mediated by RNA binding proteins and non-coding RNAs (e.g. miRNA, piRNA, lncRNA) are critical for cells to quickly adapt to growth condition changes such as nutrient stress or drug exposure. These processes are readily reversible, allowing the cell to fine tune its response to these stimuli.

miRNAs, a class of non-coding RNA, inhibit protein synthesis by perfect or imperfect base pairing with the mRNA transcript in the 3′-UTR region in conjunction with the Argonaute (Ago) complex. One particular miRNA can modulate the mRNA stability and translation rate of hundreds of mRNA target transcripts. There has been some discrepancy in our understanding of target regulation by miRNA [1]. While there have been many studies that have identified targets for which repression occurs in the absence of mRNA degradation, other studies have clearly demonstrated that mRNA degradation by miRNA is important in target repression [2-9]. David Bartel's group has shown that the majority of miRNA targets are regulated predominantly via mRNA degradation with translational repression only making minor contributions to overall regulation. In several cellular contexts mRNA degradation accounts for 66%-90% of miRNA target repression, suggesting that analysis of total steady state mRNA levels should allow for the identification of the majority of miRNA targets [10, 11] However, there may be some mRNA for which analysis of mRNA degradation alone can underestimate their repression. These targets will not be selected for further investigation and validation following high throughput screening. In such cases, translational activity analysis would be useful for identifying biologically significant miRNA targets. A good example is miR-215 which suppresses several key targets such as TS, DHFR and DTL in colon cancer. None of these targets would be selected based on steady state total RNA profiling as the degradation of these mRNA transcripts is quite small [12].

miRNA target identification based on actively translating mRNAs associated with polyribosomes has been utilized successfully [13, 14]. However, such approaches rely on traditional sucrose gradient ultracentrifugation to pool polyribosome fractions. There are several limitations of this approach. One major disadvantage is that it requires 1 × 10^7^-10^8^ cells, which is often difficult to achieve with primary cells or cancer stem cells. Recently, a photo-activatable-ribonucleoside-enhanced cross-linking and immunoprecipitation (PAR-CLIP) technique has been developed to identify miRNA-Ago complex binding sites as well as other RNA binding protein binding sites on mRNA [15, 16]. However, the degree of RNase digestion and other factors are challenges for sequencing based approaches due to variations in sequencing background. In addition, such approaches also require at least 1 × 10^7^ cells [16].

**Figure 1:**
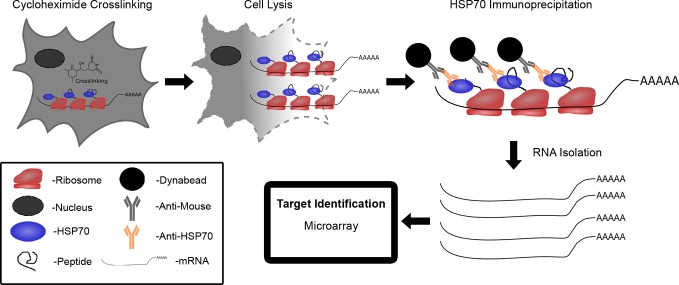
Schematic diagram of translational immunoprecipitation (TrIP-chip) approach to identify miRNA translationally regulated targets Cycloheximide treated polyribosome complexes are cross linked to mRNA via DSP. Cells are then lysed and the polyribosome/mRNA complex is immunoprecipitated by targeting HSP70 using anti-HSP70 antibody conjugated to magnetic beads. mRNA is isolated from the complex using Array Pure Nanoscale RNA Purification Kit. mRNA expression levels can then be assessed by- microarray.

In this study, we utilized a novel application of our previously developed Translational Immuno-Precipitation approach (TrIP) to discover mRNA targets regulated by miRNAs using as few as 100 cells [17]. Our approach is based on the well-established concept that newly synthesized polypeptides interact with chaperone protein HSP70s to prevent premature folding and modification [18-20]. As a result, HSP70s play a critical role in the polyribosome complex during protein synthesis. The polyribosome complex has been well documented to contain the newly synthesized polypeptide along with the mRNA and miRNA [21]. The principle of this approach is illustrated in Figure 1. This approach involves using cycloheximide to stall translation and dithio-bis (succinimidyl propionate) (DSP) to cross link the polyribosome complex to the mRNA. The polyribosome/mRNA complex is then immunoprecipitated by targeting HSP70 using an anti-HSP70 antibody conjugated to magnetic beads. HSP70 antibody affinity capture beads can isolate the translational complex containing mRNA transcripts. Following immunoprecipitation, the mRNA transcripts are isolated from the complex using the Array Pure Nano-Scale RNA Purification Kit (Epicentre) and subjected to high throughput expression analysis by microarray (chip) [17]. Changes observed in mRNA levels by microarray, in this case do not represent changes in total mRNA levels, but rather differences in active mRNA translation. One of the major advantages of this approach is that it allows us to identify, and when used in combination with expression analysis on steady-state populations, to distinguish both degraded and non-degraded miRNA targets from a small number of cells.

miR-215 was selected as a candidate for this proof-of-concept study as it is an important miRNA in colorectal cancer and its expression is regulated by tumor suppressor p53 via a positive feedback mechanism [12, 22, 23]. In addition, we have previously identified several direct targets (TYMS, DTL, DHFR) of miR-215 that are important for chemosensitivity to 5-fluorouracil treatment, cell cycle control and cell proliferation[12]. The translation of these three mRNA targets was suppressed by miR-215 with only minor mRNA degradation. As a result, these known miR-215 targets were used to monitor the effectiveness of our approach for miR-215 target identification using translational immunoprecipitation (TrIP), followed by microarray, (chip) (TrIP-chip).

**Figure 2:**
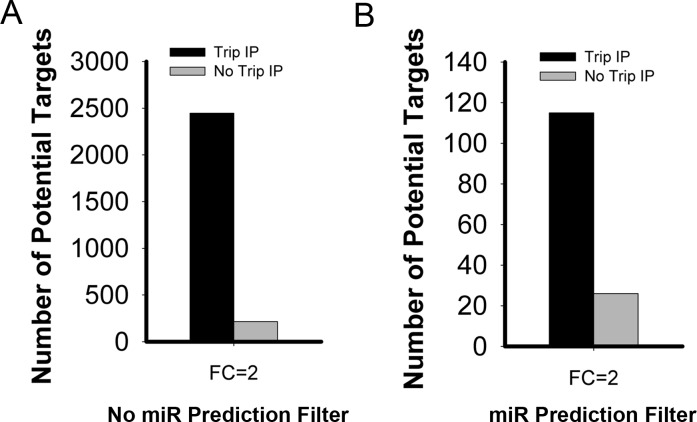
Comparative analysis of potential miR-215 mediated targets identified by TrIP-chip expression analysis *vs*. steady state total RNA based expression approach at 2-fold change cut-off **A.** Comparison solely based on experimental data without filtering with any predictive algorithms for miR-215 mediated targets (both direct and indirect targets). **B.** Comparative analysis of miR-215 direct targets after filtering with predictive algorithms. The Y axis indicates the number of potential miR-215 targets.

Using the TrIP-chip approach, we identified 2447 mRNAs with reduced active translation at 2-fold cut-off *vs.* about 215 reduced mRNA targets based on steady state total RNA profiling (Figure 2). Among the 2447 mRNA transcripts, some are identified as direct or indirect targets of miR-215. To identify direct targets of miR-215, we filtered the targets with 5 different miRNA predictive target algorithms (TargetScan, PicTar, DIANA-TarBase, miRDB, miRbase). With this approach, we have identified 116 potential direct targets of miR-215. Of these, we confirmed our ability to identified previously validated important targets that play key roles in colorectal cancer including thymidylate synthase (TS, TYMS), dihydrofolate reductase (DHFR) and denticleless (DTL), as well as novel targets such as histone H3F3B and DNA mismatch repair protein MutS homolog 6 (MSH6).

## RESULTS

### Identification of additional miR-215 mediated targets using TrIP-chip approach compared to total steady state RNA profiling

Expression profiling analysis based on total steady state RNA transcripts will miss some important miRNA targets because mRNA degradation can underestimate overall target repression. These targets would be filtered out in high throughput screening because they may fail to reach a pre-set fold change cut off. As a result, we miss important targets for further validation and investigation. When translational regulation is analyzed this fold change threshold may be reached. Using miR-215 as an example, we detected additional targets using this TrIP-chip based approach (Figure 2A). At the 2-fold cut-off, we identified 2447 potential targets of miR-215, compared to only 215 targets based on total steady state mRNA profiling.

There are several available predictive databases for identifying miRNA targets which can be valuable tools for miRNA analysis. Therefore we wanted to know if our identified targets overlapped with those predicted by these algorithms (TargetScan, PicTar, miRDB, miRbase, DIANA-TarBase). Employing this filter reduced the total number of targets identified, however we still identified more targets with our TrIP approach compared to using total steady state RNA (Figure 2B). With the most stringent filtering criteria, there was only about 6% overlap with the 2447 non-filtered targets. Based on this, we identified 116 potential direct targets of miR-215 which are shown in Supplementary Table 1.We selected a short list of genes that may be relevant in human colon cancer, shown in Table 1. Our results clearly show that we successfully captured miR-215 targets, such as TYMS, DHFR, and DTL for which mRNA degradation is limited or absent (Table 1).

**Table 1:** Selected Targets Identified By TRIP and Non-Trip Approaches

TRIP		Non-TRIP	
Target	Fold Change	Target	Fold Change
MMP16	−50.21	WNK	−12.99
ZEB2	−33.14	PHB	−2.99
CREB5	−14.91	ELMO1	−2.95
BMP1	−11.25	RUNX1T1	−2.68
H3F3B	−4.45	BMP15	−2.02
DICER1	−3.87		
DHFR	−3.49		
MCM6	−2.89		
DTL	−2.86		
TYMS	−2.81		
MSH6	−2.16		

### Validation of the TrIP-chip approach for identifying non-degraded mRNA targets of miR-215

**Figure 3:**
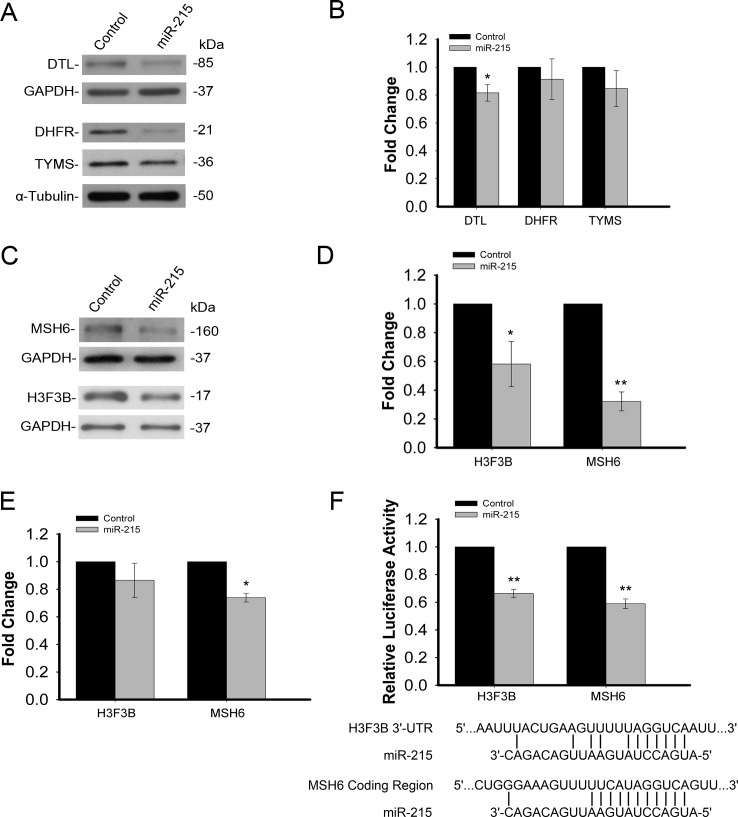
**A.** Validation of TrIP-chip approach to capture non-degraded mRNA targets based on previously known targets of miR-215 (DTL, DHFR, TYMS) by Western immunoblot analysis. **B.** qRT-PCR analysis of DTL, DHFR, TYMS expression based on total steady state RNA in response to miR-215 showing only minor, mRNA degradation. **C.** Confirmation of reduced protein expression of two novel targets (H3F3B, MSH6) of miR-215 by Western immunoblot analysis.**D.** Quantification of Western immunoblot analysis for H3F3B and MSH6, indicating a significant decrease in the expression of both proteins in response to miR-215. **E.** qRT-PCR analysis of H3F3B and MSH6 expression based on total steady state RNA in response to miR-215, indicating a slight decrease in mRNA expression. **F.** Luciferase reporter assay showing decreased luciferase activity in response to miR-215 for both H3F3B and MSH6, indicating direct targeting of these transcripts by miR-215.miR-215 binding sites are also shown for both H3F3B and MSH6. (*p < .05, **p < .001).

The effectiveness of this approach was validated using previously identified targets of miR-215, TYMS, DHFR and DTL [12, 23]. Western immunoblot analysis indicated expression at the protein level of these three targets is decreased by miR-215 (Figure 3A). However, as demonstrated previously, mRNA levels of these three targets were only slightly decreased (Figure 3B). We also selected some important targets that may play key roles in colorectal cancer for validation namely, MSH6 and histone H3F3B. These two targets were confirmed to be reduced in response to miR-215 by Western immunoblot analysis (Figure 3C) and quantification indicated this reduction was highly significant (Figure 3D). The significant decrease in protein expression is consistent with the TrIP-chip data in Table 1. The mRNA levels of MSH6 and H3F3B based on the total steady state RNAs were quantified by real time qRT-PCR analysis (Figure 3E). While both target mRNA transcripts decreased slightly, these small differences would likely be filtered out and the targets not selected for further investigation during high throughput expression analysis. In contrast, the decrease in translational activity quantified by TrIP-chip expression analysis (Table 1) and protein levels were significant. To further verify that MSH6 and Histone H3F3B are direct targets of miR-215, we cloned the 3′-UTR region of Histone H3F3B and a potential binding site of miR-215 in the MSH6 protein coding region into a luciferase reporter. The potential binding site in the coding region of MSH6 was identified by a BLAST search which revealed complementarity between this region and miR-215, including the seed region. Our results show that miR-215 is able to reduce luciferase activity for both targets while the control miRNA has no effect on luciferase activity (Figure 3F).

### Pathway analysis mediated by miR-215

**Figure 4:**
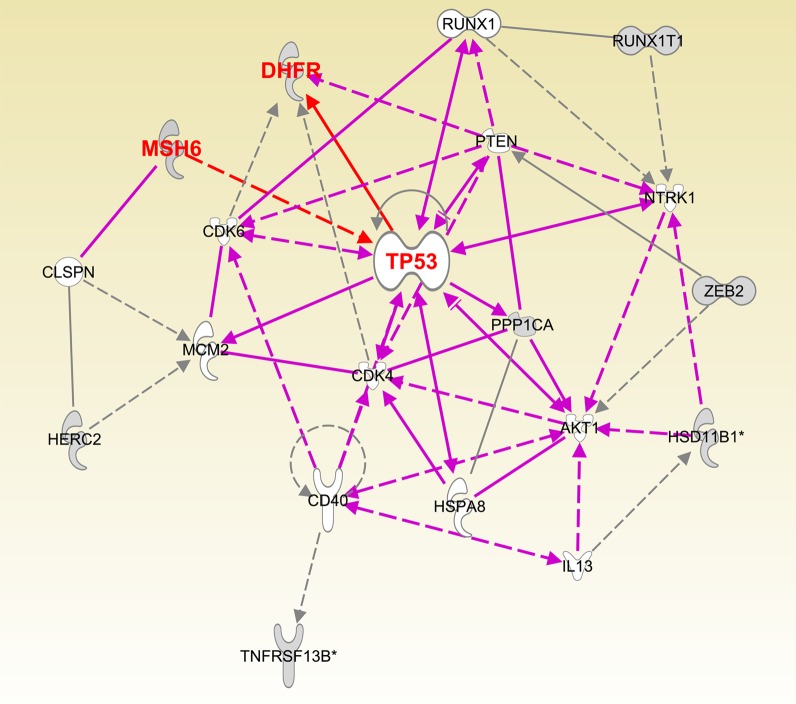
Pathway analysis of major targets of miR-215 determined by Ingenuity Pathway Analysis software

To systematically determine the pathways regulated by miR-215, we utilized Gene Ontology analysis to reveal the pathways and targets impacted by miR-215. Figure 4 and Supplementary Figure 1 show the major target networks mediated by miR-215. miR-215 forms a positive feedback loop with tumor suppressor gene p53 to exert its tumor suppressive function by targeting DHFR and mismatch repair gene MSH6 (Supplementary Figure 1). A more focused view of top 20 targets and pathways revealed that miR-215 also impacts EMT gene Zeb2 and the cell cycle control genes CDK4, CDK6, PTEN and AKT (Figure 4). Gene Ontology analysis revealed that PI3K, Oct4 stem cell pluripotency, DNA damage response and G1/S check point control pathways are major networks influenced by miR-215 (Supplementary Figure 2).

## DISCUSSION

In this study, we utilized a novel application of the TrIP-chip approach to identify miRNA targets and pathways from a small number of cells. This approach gives us a comprehensive overview of both miR-215 direct and indirect targets, and the pathways that miR-215 impacts in colon cancer. In addition to colon cancer, miR-215 has been shown to be an important miRNA in other types of cancer. However, our understanding of how miR-215 contributes to this disease is still rather limited. The new approach described in this study provides a broader view of how miR-215 may contribute to regulate cell growth, the cell cycle, and chemosensitivity.

It has been recently demonstrated in *C. elegans* that changes in mRNA abundance and ribosome loading regulated by miRNA let-7 affects only a very small portion of targets [24]. The ribosome occupancy profiles do not change dramatically on miRNA-repressed mRNAs. The activity of the translational machinery is an important part of miRNA mediated gene regulation. This supports the notion that profiling mRNA transcripts by isolating translationally active mRNA polyribosome complexes will likely provide insight into how miRNAs regulate translation of their targets. Work by David Bartel's group has demonstrated that the majority (66%-90%) of miRNA regulation may be the result of mRNA degradation, While this would suggest that analysis of total steady state mRNA will identify the majority of miRNA targets, the TrIP approach is useful for identifying those potentially important targets for which translational regulation may be important [10, 11]. These mRNAs would be missed in high throughput analysis because their fold change does not reach a specified cut off when only considering steady state mRNA levels. When taking into account translational regulation these targets can be identified and selected for further functional investigations. In addition, this approach can identify both degraded and non-degraded targets, making it useful for extensive miRNA target identification. The functional effects of miRNA regulation are ultimately changes in protein level, and thus this approach to analyze translational activity more closely measures the functional output of miRNA regulation without the need for proteomics analysis.

To identify miR-215 mediated targets in colon cancer, we filtered the miR-215 targets using current available bioinformatics algorithms. We realize that while very useful, such a filter is limited and the predictive bioinformatics algorithms can have a high range of false positive rates [16, 25, 26]. With this in mind, we identified about 120 mRNA targets mediated by miR-215. Analysis of previous identified miR-215 targets such as TYMS, DHFR, and DTL, allowed us to monitor and to validate the effectiveness of this new approach (Table 1, Figure 3A and 3B). It is interesting to note that DTL is not a predicted target of miR-215 by all five available predictive algorithms. However, our results show that DTL is a direct target of miR-215 [12]. This reflects the intrinsically difficult nature of predicting miRNA targets as mRNA can fold into different secondary and/or tertiary structures with various RNA binding proteins and such complex structures are highly dynamic.

Several sequencing based approaches (Ago-CLIP, HITS-CLIP) have been developed to investigate protein/mRNA or miRNA/mRNA interactions [27, 28]. These approaches have provided unique tools for identifying miRNA targets. However, just as with any other approach, there are a number of issues that still remain to be improved. These include the quality of the antibody used for immunoprecipitation, the degree of RNAse treatment, and inherent biases and artifacts from PCR and small RNA sequencing library preparation. Our TrIP-chip approach for miRNA target identification will capture both degraded and non-degraded mRNA targets. Degraded targets, by nature of their decreased representation in the mRNA pool, will have decreased association with active translation complexes. In addition, this approach can identify those targets that are directly regulated by a miRNA as well as downstream targets whose translational activity is altered. This can be greatly advantageous in identifying the larger cellular pathways that a miRNA may regulate. It can also be used for a small number of cells given its high level of sensitivity. Although the TrIP-chip approach will not be able to directly identify miRNA target binding sites, it provides a sensitive platform to identify changes in active mRNA translation. This approach provides some unique insights compared to Ago-CLIP, as it identifies mRNA targets whose translation is being inhibited by a particular miRNA rather than just binding partners, and thus measures more of a functional output. Our TrIP-chip approach and the Ago-CLIP approach, could complement each other quite well for a thorough investigation of a miRNA's targets. Ago-CLIP would indicate binding sites on targets while the TrIP-chip approach will demonstrate those targets whose translation is actually being altered. The TrIP-chip approach is clearly an improvement compared to traditional sucrose gradient ultracentrifugation which uses a large number of cells. It provides a quantitative measure of miR-215 target expression at a greater depth and sensitivity than proteomics analysis.

We further validated several new targets (histone H3F3B and MSH6) of miR-215 based on their potential biological significance in colorectal cancer [29, 30]. MSH6 is a key protein involved in DNA mismatch repair and works in conjunction with MSH2 [31]. Disruption of this gene is associated with Lynch syndrome [32]. This link to colon cancer makes the inhibition of this gene by miR-215 particularly interesting. It also demonstrates that miR-215 can impact important cellular pathways such as DNA repair. Furthermore, the luciferase assay performed in this study indicated that miR-215 targets MSH6 not in the 3′-UTR but rather in the coding region of the mRNA transcript. This type of regulation is missed by prediction algorithms. Other studies have shown 25% of miRNA targets are targeted in their coding regions, demonstrating that this is an important aspect of miRNA function [16]. Histone H3 is one of 4 proteins that make up the histone core. There are 19 known genes that code for H3 isoforms [33]. While some of these isoforms are considered canonical and their production is coordinated with S phase, there are other isoforms that are produced throughout the cell cycle and replace the canonical isoforms in certain situations. H3F3B codes for an H3.3 isoform, and its expression is not tied to S phase. Unlike the canonical H3 isoforms, H3F3B has introns and a poly-A tail, distinguishing its post transcriptional processing from that of the canonical H3 isoforms [33]. In contrast to other H3 mRNAs, which have short (50 bp) 3′-UTR's, H3F3B has a long (2162 bp) 3′-UTR allowing it to be regulated by miRNAs targeting the 3′-UTR. The H3.3 isoform has been associated with active transcription, however it is also found in non-transcribed regions such as centromeres and telomeres [34, 35]. Thus the exact function of this isoform remains to be characterized. It will be interesting to determine the significance of miR-215 regulation of this protein, as well as what role this may play in colon cancer. Suppression of H3F3B by miR-215 may affect transcription of other genes, enhancing the global effect of miR-215 in cancer cells. Another potentially important target, MMP16, has been shown to be increased in stem like colon cancer cells [36]. This protein is also associated with invasion and migration in glioma cells and exhibits differential expression in differentiating colon cancer cells [37, 38]. It will be of great interest to determine if miR-215 plays a role in the regulation of MMP16 in these various conditions. In addition, the regulation of Zeb2 by miR-192 may reduce metastasis in colon cancer [39]. It will be interesting to determine if miR-215 is involved in this regulation, as well as to understand which miRNA has a more prominent role in Zeb2 regulation *in vivo*.

We also tried to elucidate the target network and pathways impacted by miR-215 using Ingenuity Pathway Analysis software. The target network allows us to visualize the connections between key targets and their associations with the phenotypic impact of miR-215 on proliferation, cell cycle control, and cell death (Figure 4). The network also reflects our previous knowledge of the participation of miR-215 and its transcription factor p53 in a positive feedback loop to regulate the cell cycle [12, 23]. Our results support the notion that miR-215 exerts its functions by influencing several key targets and pathways. The functional significance of miR-215 in regulating MSH6, histone H3F3B and other important targets will be investigated in future studies.

HSP70s are the major class of chaperones that shield the hydrophobic regions of nascent and incompletely folded polypeptides from premature folding, modifications and intermolecular interactions. We have demonstrated that the TrIP-chip approach can capture all major post-transcriptional and translational events regulated by RNA binding proteins [17]. There is still a possibility that our approach may not be able to capture all miR-215 targets. With regards to the detection sensitivity of current microarray expression analysis, we feel that the expression analysis can be further improved using a next generation sequencing platform and the cell numbers required can be further reduced to the single cell level (TrIP-seq). This approach can be used along with other developed approaches to discover and cross validate miRNA mediated targets.

In conclusion, we developed a universal method to systematically identify miRNA mediated targets and pathways. Our TrIP-chip approach, much like the ChIP-chip approach for interrogating transcriptional regulation, has the potential to be a useful tool to investigate both post-transcriptional and translational control mediated by miRNA and other classes of non-coding RNAs, and RNA binding proteins.

## MATERIALS AND METHODS

### Cell lines and transfection

The human colon cancer cell line HCT-116 (wt-p53) was obtained from the American Type Culture Collection (ATCC) and maintained in McCoy's 5A medium (Gibco Laboratories). Media was supplemented with 10% fetal bovine serum (HyClone Laboratories). Transfection was carried out as optimized previously [12]. Briefly, HCT-116 (wt-p53) cells (2×10^5^) were plated in six-well plates and transfected with 100 nM of either miR-215 precursor or non-specific miRNA (Invitrogen) after 24 hours by Oligofectamine (Invitrogen) according to the manufacturer's protocols.

### Translational complex immunoprecipitation (TrIP-chip)

The construction of the antibody affinity capture magnetic beads was carried out as previously reported [17]. In brief, the required amount of thoroughly suspended beads was transferred into an Eppendorf tube (Dynabeads®M-280 Sheep anti-Mouse IgG). Beads were washed two times with washing buffer (PBS with 0.1% BSA, pH 7.4) for Dynabeads^®^M-280 Sheep anti-Mouse IgG). Then 1μg/μl target immunoglobulin was added at a ratio of 1 μg/10^7^ beads. HSP70 mouse monoclonal antibody (Millipore Inc.) was used for bead conjugation. Beads with antibody were incubated with slow tilt rotation for 2 hours at 4°C. The tube was then placed on a magnetic stand for 2 minutes and supernatant was removed with a pipette. Antibody-coated beads were washed three times with washing buffer and used for immunoprecipitation.

### Isolation of polysome associated mRNA transcripts

To prepare cytoplasmic extracts, based on previous optimizations [12], 48 hours after transfection, control colon cancer HCT-116 cells and cells transfected with 100 nM miR-215 from 6-well tissue culture plates were washed with ice-cold PBS containing 100 μg/ml cycloheximide (Sigma) and harvested with 0.05% Trypsin-EDTA (Invitrogen). Cells were counted and 100-100,000 cells were incubated with 1600 μl of McCoy's 5A medium containing 10% fetal bovine serum and 100 μg/ml cycloheximide (Sigma) for 5 min at 37°C. Following incubation, 400 μl of DSP (1 mM) (Pierce) was introduced as a cross-linking reagent and incubation was carried out for 5 min at 37°C. Excess DSP was quenched with 1 M Tris-HCl (pH 7.4). The cells were washed twice by centrifugation at 1,500 rpm for 5 min, the supernatant discarded, and the cell pellets were rinsed twice with ice-cold PBS containing 100 μg/ml cycloheximide (Sigma). The final pellets were swollen for 20 min in 1000 μl of low salt buffer (LSB) (20 mM HEPES, pH 7.4, 100 mM KCL, 2 mM MgCl_2_) containing 1 mM dithiothreitol and lysed by the addition of 400 μl lysis buffer (1× LSB containing 1.2% Triton X-100) (Sigma) followed by brief vortexing. The above lysate was transferred to the anti-HSP70 coated beads, and incubation was carried out for 4 hours at 4°C with slow tilt rotation.

After incubation with the HSP70 antibody conjugated magnetic beads, the polysome complexes containing translationally active mRNA transcripts were isolated, and the mRNAs were eluted from the beads using the Array Pure Nanoscale RNA Purification Kit (Epicentre).

### Quantitative gene expression analysis via real-time quantitative reverse transcription-PCR

Real-time quantitative reverse transcription-PCR (qRT-PCR) analysis was performed on non-TrIP-chip mRNAs, isolated from HCT-116 cells transfected with miR-215 or non specific miRNA precursor. TaqMan-PCR primer/probes (TYMS, DHFR, MSH6, DTL, histone H3F3B, GAPDH) were purchased from Life Technologies. qRT-PCR was performed on an ABI 7500HT instrument under the following conditions: 25°C for 10 min and 37°C for 30 min of reverse transcription; 95° for 3 min, followed by 40 cycles of 95°C for 15 sec and 60°C for 35 sec (*n* = 3). Signal was collected at the endpoint of every cycle. The expression values of genes from different samples were calculated by normalizing with housekeeping gene GAPDH and relative quantification values were plotted.

### Gene expression analysis

All reagents were provided in the Gene Expression Hybridization Kit (Agilent). 11 μl of 10 x Blocking Agent and nuclease-free water was added to 1.65 μg of Cy3-labeled linearly amplified cRNA to bring the volume to 53 μl. Then 2.2 μl of 25x Fragmentation Buffer was added. After vortexing, the RNAs were fragmented by incubating at 60 °C for 30 min. To prepare hybridization mix, 55 μl of fragmented cRNA and 55 μl of 2 x GE Hybridization Buffer HI-RPM were mixed and carefully loaded onto the array gasket well to avoid bubbles. Hybridization was carried out at 10 rpm at 65 °C for 17 hours in a hybridization oven. After hybridization, the arrays were washed with Gene Expression Wash Buffer 1 (Agilent). The slides were then dried and kept in the dark until scanning. Images were captured on an Axon GenePix 4200A scanner.

### Target identification of miR215 via an integrated algorithms

A heuristic algorithm comprised of both data-driven and knowledge driven filters was developed to identify the putative miR-215 targets for both the TrIP-chip and non-TrIP-chip approaches. In both settings, the RNA expression profiles of 22181 genes were investigated using Agilent microarray analysis with control samples and miR-215 transfected samples. All experiments were performed in triplicate. In each analysis, a preliminary filter was applied to remove probes having a value < 1 in 80% of the samples, subsequently a general scaling method [40] was applied to remove between group variances, ensuring comparable downstream analysis. Due to the small number of samples, an exploratory data-driven filter based on FC (fold change) was applied to identify gene probes negatively modulated by miR-215 (FC > 2) in paired comparison. In the following step, we implemented a knowledge driven filter to further narrow down the putative targets from the last step; this filter was comprised of 5 established miRNA target prediction algorithms including miRbase [41], miRDB [42], PicTar [43], TargetScan [44] and DIANA-TarBase [45]. A putative gene target identified from the data-driven filter can be considered a probable target once it is confirmed in any of the 5 prediction algorithms (knowledge-driven filter).

### Protein confirmation via western immunoblot analysis

Equal amounts of protein (15μg) extracted from cells lysed in RIPA buffer with protease inhibitor (Sigma) were separated on 10%-12% sodium dodecyl sulfate-polyacrylamide gels by the method of Laemmli [46]. Proteins were probed with mouse anti-TYMS monoclonal antibody (1:400 dilution) (Zymed Laboratories), anti-MSH6 (1:1000) (Cell Signaling Technology), anti-histone H3 (1:20000) (Bethyl Laboratories Inc.), anti-DHFR mouse monoclonal antibody (1:250) (Santa Cruz Biotech Inc.), anti-α-tubulin (1:20000) (Santa Cruz Biotech Inc.), anti-GAPDH (1:20000) (Santa Cruz Biotech Inc.) and anti-DTL (1:400) (Santa Cruz Biotech Inc.). Horseradish peroxidase–conjugated antibodies against mouse or rabbit (1:5000, Santa Cruz Biotech Inc.) were used as the secondary antibodies. Protein bands were visualized with autoradiography film using SuperSignal West Pico Chemiluminescent Substrate (Thermo).

### Luciferase reporter assay

A 618 nucleotide portion of the MSH6 mRNA coding region was determined to contain a sequence complementary to the seed sequence of miR-215 and was cloned into pMIR-Report Vector (Life Technologies) using forward primer: CGAGCTCGCACGAGTGGAACAG and reverse primer: CGACGCGTACCACCTAGAGCAGA. A 1425 nucleotide portion of the H3F3B 3′-UTR which was predicted to be targeted by miR-215 was also cloned into pMIR-Report Vector using forward primer: CGACGCGTGTGAAGGCAGTTTTT, and reverse primer: CGCGCCGTTTAAACCTGAGTTCTACACCT. Twenty-four hours before transfection, 1.5 × 10^4^ cells were plated in a 96-well plate. 10 nM of miR-215 or control miRNA was transfected into these cells together with 100 ng of pMIR-Report-MSH6 or pMIR-Report-H3F3B and 1 ng of Renilla luciferase plasmid pRL-SV40 (Promega) with DharmaFect Duo (Dharmacon) following the manufacturer's protocol. The luciferase assay was performed 24 h after transfection with the dual-luciferase reporter assay system (Promega). For each sample, firefly luciferase activity was normalized to Renilla luciferase activity and the inhibition by miR-215 was normalized to the control miRNA and repeated 3 times.

### Statistical analysis

All experiments were repeated at least three times. All statistical analyses were performed using SigmaPlot software. Statistical significance between two groups was determined using Student's *t*-test. (*p < .05, **p < .001)

## SUPPLEMENTARY MATERIAL FIGURES AND TABLES



## References

[1] Wilczynska A, Bushell M (2015). The complexity of miRNA-mediated repression. Cell death and differentiation.

[2] Olsen PH, Ambros V (1999). The lin-4 regulatory RNA controls developmental timing in Caenorhabditis elegans by blocking LIN-14 protein synthesis after the initiation of translation. Developmental biology.

[3] Seggerson K, Tang L, Moss EG (2002). Two genetic circuits repress the Caenorhabditis elegans heterochronic gene lin-28 after translation initiation. Developmental biology.

[4] Poy MN, Eliasson L, Krutzfeldt J, Kuwajima S, Ma X, Macdonald PE, Pfeffer S, Tuschl T, Rajewsky N, Rorsman P, Stoffel M (2004). A pancreatic islet-specific microRNA regulates insulin secretion. Nature.

[5] Bandyopadhyay S, Long ME, Allen LA (2014). Differential Expression of microRNAs in Francisella tularensis-Infected Human Macrophages: miR-155-Dependent Downregulation of MyD88 Inhibits the Inflammatory Response. PloS one.

[6] Chen JF, Mandel EM, Thomson JM, Wu Q, Callis TE, Hammond SM, Conlon FL, Wang DZ (2006). The role of microRNA-1 and microRNA-133 in skeletal muscle proliferation and differentiation. Nature genetics.

[7] Chen CY, Zheng D, Xia Z, Shyu AB (2009). Ago-TNRC6 triggers microRNA-mediated decay by promoting two deadenylation steps. Nature structural & molecular biology.

[8] Zhai H, Song B, Xu X, Zhu W, Ju J (2013). Inhibition of autophagy and tumor growth in colon cancer by miR-502. Oncogene.

[9] Rehwinkel J, Behm-Ansmant I, Gatfield D, Izaurralde E (2005). A crucial role for GW182 and the DCP1:DCP2 decapping complex in miRNA-mediated gene silencing. Rna.

[10] Eichhorn SW, Guo H, McGeary SE, Rodriguez-Mias RA, Shin C, Baek D, Hsu SH, Ghoshal K, Villen J, Bartel DP (2014). mRNA Destabilization Is the Dominant Effect of Mammalian MicroRNAs by the Time Substantial Repression Ensues. Molecular cell.

[11] Guo H, Ingolia NT, Weissman JS, Bartel DP (2010). Mammalian microRNAs predominantly act to decrease target mRNA levels. Nature.

[12] Song B, Wang Y, Titmus MA, Botchkina G, Formentini A, Kornmann M, Ju J (2010). Molecular mechanism of chemoresistance by miR-215 in osteosarcoma and colon cancer cells. Molecular cancer.

[13] Arava Y, Wang Y, Storey JD, Liu CL, Brown PO, Herschlag D (2003). Genome-wide analysis of mRNA translation profiles in Saccharomyces cerevisiae. Proceedings of the National Academy of Sciences of the United States of America.

[14] Ju J, Huang C, Minskoff SA, Mayotte JE, Taillon BE, Simons JF (2003). Simultaneous gene expression analysis of steady-state and actively translated mRNA populations from osteosarcoma MG-63 cells in response to IL-1alpha via an open expression analysis platform. Nucleic acids research.

[15] Hafner M, Landthaler M, Burger L, Khorshid M, Hausser J, Berninger P, Rothballer A, Ascano M, Jungkamp AC, Munschauer M, Ulrich A, Wardle GS, Dewell S, Zavolan M, Tuschl T (2010). Transcriptome-wide identification of RNA-binding protein and microRNA target sites by PAR-CLIP. Cell.

[16] Chi SW, Zang JB, Mele A, Darnell RB (2009). Argonaute HITS-CLIP decodes microRNA-mRNA interaction maps. Nature.

[17] Kudo K, Xi Y, Wang Y, Song B, Chu E, Ju J, Russo JJ, Ju J (2010). Translational control analysis by translationally active RNA capture/microarray analysis (TrIP-Chip). Nucleic acids research.

[18] Anfinsen CB (1973). Principles that govern the folding of protein chains. Science.

[19] Georgopoulos C, Welch WJ (1993). Role of the major heat shock proteins as molecular chaperones. Annual review of cell biology.

[20] Hartl FU (1996). Molecular chaperones in cellular protein folding. Nature.

[21] Maroney PA, Yu Y, Fisher J, Nilsen TW (2006). Evidence that microRNAs are associated with translating messenger RNAs in human cells. Nature structural & molecular biology.

[22] Song B, Ju J (2010). Impact of miRNAs in gastrointestinal cancer diagnosis and prognosis. Expert reviews in molecular medicine.

[23] Song B, Wang Y, Kudo K, Gavin EJ, Xi Y, Ju J (2008). miR-192 Regulates dihydrofolate reductase and cellular proliferation through the p53-microRNA circuit. Clinical cancer research : an official journal of the American Association for Cancer Research.

[24] Stadler M, Artiles K, Pak J, Fire A (2012). Contributions of mRNA abundance, ribosome loading, and post- or peri-translational effects to temporal repression of C. elegans heterochronic miRNA targets. Genome research.

[25] Rajewsky N (2006). microRNA target predictions in animals. Nature genetics.

[26] Baek D, Villen J, Shin C, Camargo FD, Gygi SP, Bartel DP (2008). The impact of microRNAs on protein output. Nature.

[27] Licatalosi DD, Mele A, Fak JJ, Ule J, Kayikci M, Chi SW, Clark TA, Schweitzer AC, Blume JE, Wang X, Darnell JC, Darnell RB (2008). HITS-CLIP yields genome-wide insights into brain alternative RNA processing. Nature.

[28] Darnell RB (2010). HITS-CLIP: panoramic views of protein-RNA regulation in living cells. Wiley interdisciplinary reviews RNA.

[29] Miyoshi N, Ishii H, Nagai K, Hoshino H, Mimori K, Tanaka F, Nagano H, Sekimoto M, Doki Y, Mori M (2010). Defined factors induce reprogramming of gastrointestinal cancer cells. Proceedings of the National Academy of Sciences of the United States of America.

[30] Miyaki M, Konishi M, Tanaka K, Kikuchi-Yanoshita R, Muraoka M, Yasuno M, Igari T, Koike M, Chiba M, Mori T (1997). Germline mutation of MSH6 as the cause of hereditary nonpolyposis colorectal cancer. Nature genetics.

[31] Edelbrock MA, Kaliyaperumal S, Williams KJ (2013). Structural, molecular and cellular functions of MSH2 and MSH6 during DNA mismatch repair, damage signaling and other noncanonical activities. Mutation research.

[32] Peltomaki P (2014). Epigenetic mechanisms in the pathogenesis of Lynch syndrome. Clinical genetics.

[33] Ederveen TH, Mandemaker IK, Logie C (2011). The human histone H3 complement anno 2011. Biochimica et biophysica acta.

[34] Szenker E, Ray-Gallet D, Almouzni G (2011). The double face of the histone variant H3.3. Cell research.

[35] Talbert PB, Henikoff S (2010). Histone variants--ancient wrap artists of the epigenome. Nature reviews Molecular cell biology.

[36] Xu XT, Xu Q, Tong JL, Zhu MM, Nie F, Chen X, Xiao SD, Ran ZH (2012). MicroRNA expression profiling identifies miR-328 regulates cancer stem cell-like SP cells in colorectal cancer. British journal of cancer.

[37] Li Y, Wang Y, Yu L, Sun C, Cheng D, Yu S, Wang Q, Yan Y, Kang C, Jin S, An T, Shi C, Xu J, Wei C, Liu J, Sun J (2013). miR-146b-5p inhibits glioma migration and invasion by targeting MMP16. Cancer letters.

[38] Astarci E, Erson-Bensan AE, Banerjee S (2012). Matrix metalloprotease 16 expression is downregulated by microRNA-146a in spontaneously differentiating Caco-2 cells. Development, growth & differentiation.

[39] Geng L, Chaudhuri A, Talmon G, Wisecarver JL, Are C, Brattain M, Wang J (2014). MicroRNA-192 suppresses liver metastasis of colon cancer. Oncogene.

[40] Yang YH, Dudoit S, Luu P, Lin DM, Peng V, Ngai J, Speed TP (2002). Normalization for cDNA microarray data: a robust composite method addressing single and multiple slide systematic variation. Nucleic acids research.

[41] Ambros V, Bartel B, Bartel DP, Burge CB, Carrington JC, Chen X, Dreyfuss G, Eddy SR, Griffiths-Jones S, Marshall M, Matzke M, Ruvkun G, Tuschl T (2003). A uniform system for microRNA annotation. Rna.

[42] Wang X, El Naqa IM (2008). Prediction of both conserved and nonconserved microRNA targets in animals. Bioinformatics.

[43] Krek A, Grun D, Poy MN, Wolf R, Rosenberg L, Epstein EJ, MacMenamin P, da Piedade I, Gunsalus KC, Stoffel M, Rajewsky N (2005). Combinatorial microRNA target predictions. Nature genetics.

[44] Lewis BP, Burge CB, Bartel DP (2005). Conserved seed pairing, often flanked by adenosines, indicates that thousands of human genes are microRNA targets. Cell.

[45] Vergoulis T, Vlachos IS, Alexiou P, Georgakilas G, Maragkakis M, Reczko M, Gerangelos S, Koziris N, Dalamagas T, Hatzigeorgiou AG (2012). TarBase 6.0: capturing the exponential growth of miRNA targets with experimental support. Nucleic acids research.

[46] Laemmli UK (1970). Cleavage of structural proteins during the assembly of the head of bacteriophage T4. Nature.

