# Supplementary material for: Identification of miR-215 mediated targets/pathways via translational immunoprecipitation expression analysis (TrIP-chip)
Source: Oncotarget. 2015 Jun 10;6(27):24463–73. doi: 10.18632/oncotarget.4425 (PMC4695198; doi:10.18632/oncotarget.4425)
Supplement: Supplementary file 1 [file oncotarget-06-24463-s001.pdf]

Identification of miR-215 mediated targets/pathways via translational immunoprecipitation expression analysis (TrIP-chip) approach

Supplementary Material

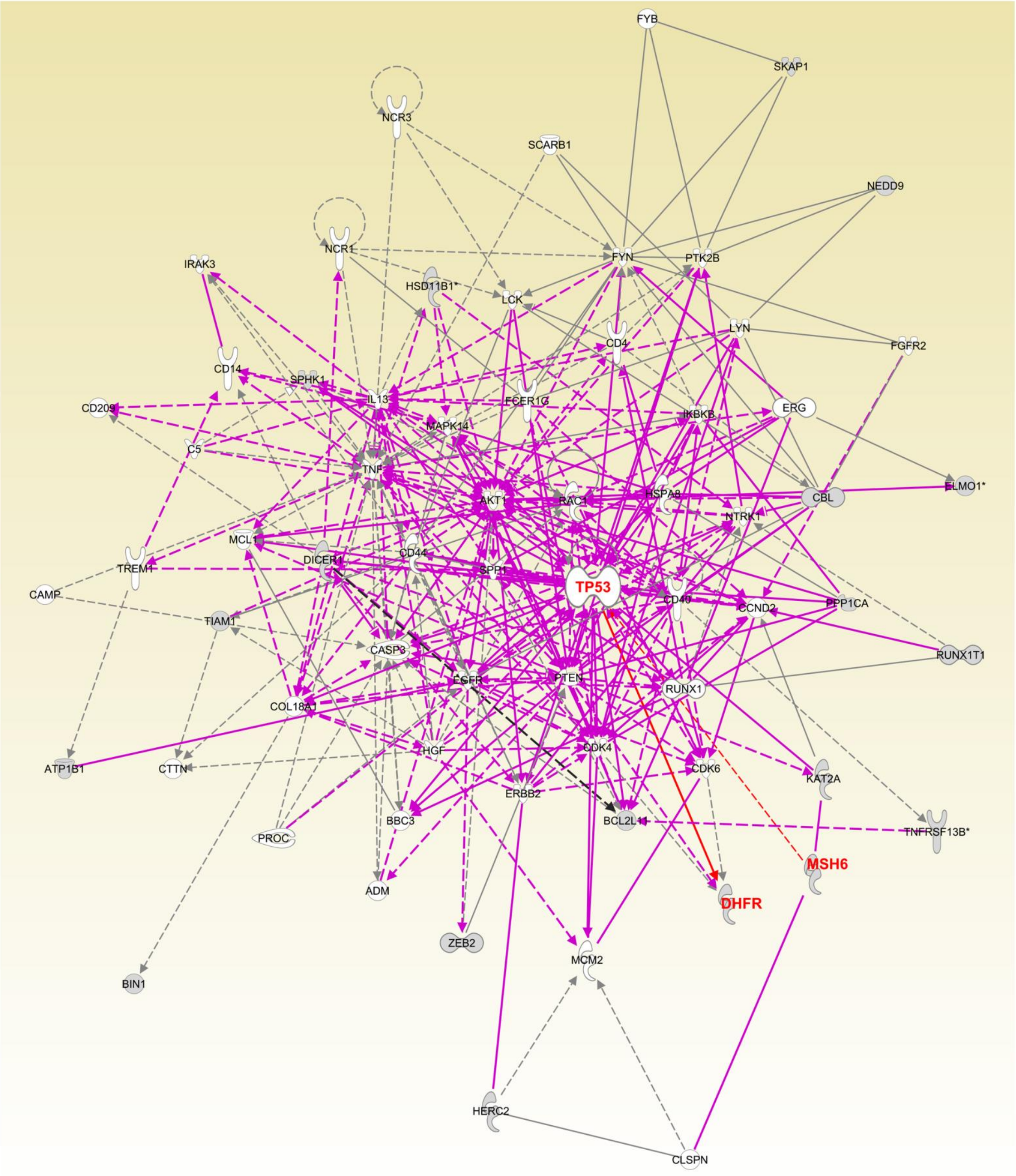

Supplementary Figure 1: Major target networks regulated by miR-215 determined by Ingenuity Pathway Analysis software.

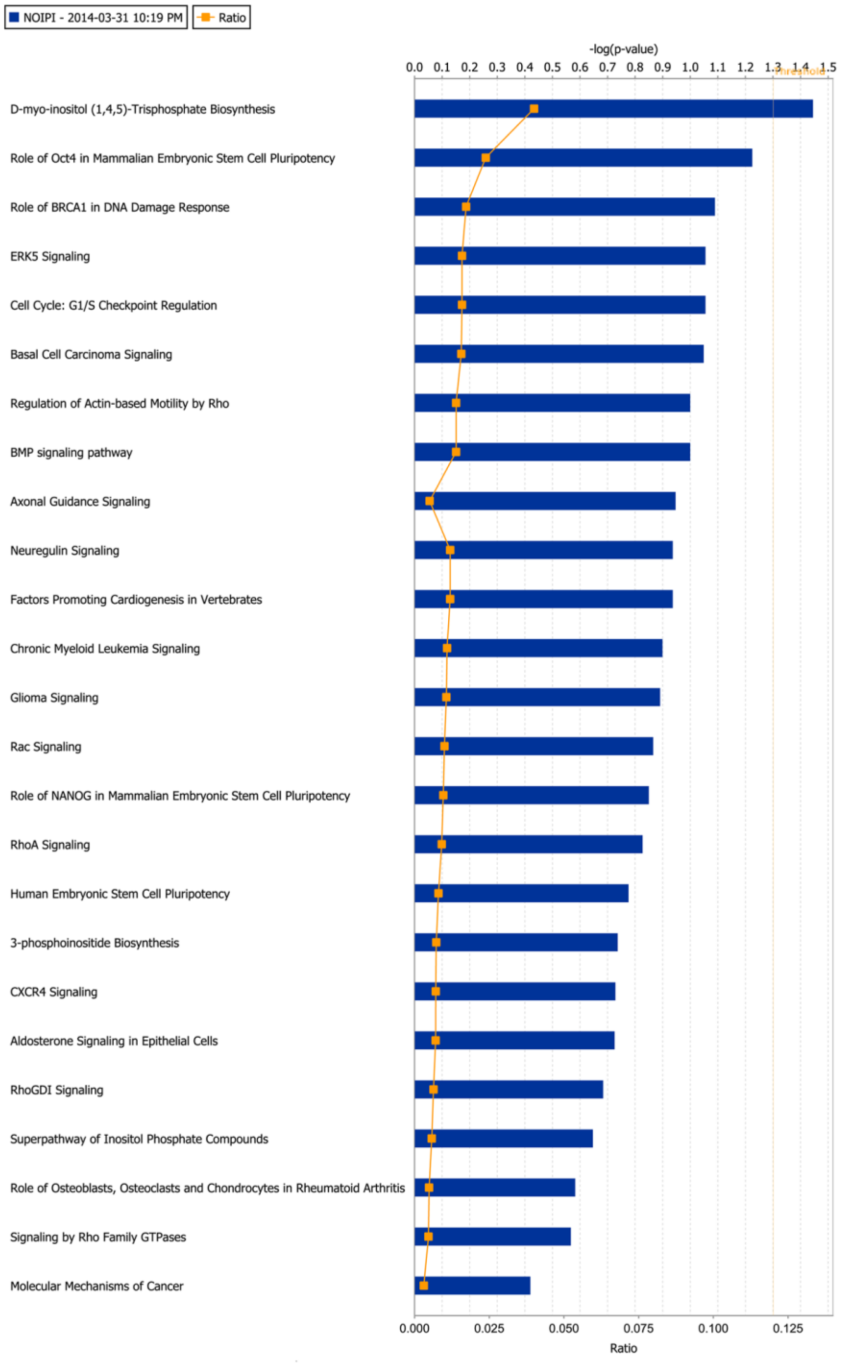

Supplementary Figure 2: Top major cellular pathways regulated by miR-215 determined by Gene Ontology analysis.

Supplementary Table 1: miR-215 Targets Identified By TRIP and Non-Trip Approaches

TRIP		Non-TRIP	
Target	Fold Change	Target	Fold Change
FAM155A	-130.94	WNK	-12.99
LEKR1	-114.15	RNF4	-12.68
OR51V1	-72.26	TMTC3	-5.27
DMBT1	-52.65	FAM155A	-4.82
UGT2B28	-50.65	PAPPA2	-4.1
MMP16	-50.21	PRM3	-3.43
NELL1	-49.07	HSD11B1	-3.41
ZEB2	-33.14	FTHL17	-3.19
VPREB3	-23.8	AMER3	-2.99
SH2D6	-23.56	PHB	-2.99
OR8D2	-20.6	ELMO1	-2.95
WFDC13	-18.74	PDZD3	-2.95
KCNC3	-18.06	LRRC10B	-2.95
CARS2	-17.1	ARPP21	-2.84
CREB5	-14.91	KATNAL2	-2.78
BCAT1	-14.76	RUNX1T1	-2.68
CIB2	-14.51	OSBPL6	-2.68
TCL6	-14.34	C12orf63	-2.51
LOXL2	-13.33	LPHN3	-2.37
TRMT61A	-13.25	SRRM4	-2.37
CUX1	-12.05	KRT72	-2.34
BIN1	-12	BMP15	-2.02
GUCY1A2	-11.91	ALG10B	-1.82
BMP1	-11.25	ANKRD44	-1.75
KAT2A	-10.5	PI4KA	-1.66
CGREF1	-10.28	RBL2	-1.54
PI4KA	-10.26		
CCDC171	-10.21		
OR4S1	-10.1		
PCNXL2	-10.1		
OR2L2	-9.98		
NEDD9	-9.54		
SH3D21	-9.43		
MTMR12	-9.07		
HSD11B1	-8.77		
RNF6	-8.68		
TRAIP	-8.19		
ELMO1	-8.18		

SLC26A2	-7.81		
DLGAP4	-7.78		
SEC13	-7.67		
SLITRK4	-7.6		
GNG3	-7.46		
ACVR2B	-7.36		
SPHK1	-7.31		
SLC17A3	-6.96		
RUNX1T1	-6.96		
ZSCAN20	-6.86		
ZNF135	-6.73		
VSX1	-6.44		
PDE2A	-6.27		
WNK1	-6.03		
TIAM1	-5.91		
ATG16L2	-5.81		
MLL3	-5.64		
HERC2	-5.53		
UPF3B	-5.51		
PPP2CB	-5.49		
MED14	-5.46		
MSRB2	-5.25		
STK32A	-5.21		
ERCC3	-5.11		
NA	-5.09		
KCNG1	-5.08		
PCIF1	-5.07		
KRT35	-4.85		
FAM134A	-4.72		
KCNIP2	-4.7		
TNFRSF13B	-4.65		
H3F3B	-4.45		
ASXL2	-4.2		
MED28	-4.2		
KPNA6	-3.94		
SYT15	-3.88		
DICER1	-3.87		
CISD1	-3.56		
DDX50	-3.54		
DHFR	-3.49		
TECPR1	-3.48		
FOXN1	-3.45		

AMER3	-3.44		
JAGN1	-3.14		
PIP4K2B	-3.14		
PAPPA2	-3.08		
MCTS1	-3.03		
RNF141	-2.99		
C21orf67	-2.96		
GRHL1	-2.95		
ATP1B1	-2.94		
CPSF4	-2.91		
MCM6	-2.89		
CBL	-2.87		
DTL	-2.86		
PPP1CA	-2.84		
TYMS	-2.81		
APBB1	-2.8		
DCAF4L2	-2.8		
NOB1	-2.77		
GABPB1	-2.75		
ELOVL1	-2.7		
COPA	-2.68		
SLC2A8	-2.61		
KIF1B	-2.6		
FGD5	-2.52		
SMARCA5	-2.48		
FAM20A	-2.47		
ODC1	-2.42		
OLIG2	-2.38		
TTC21B	-2.29		
NOC4L	-2.23		
MSH6	-2.16		
SKAP1	-2.11		
BCL2L11	-2.11		
CDON	-2.01		
DDX55	-1.98		
SH3TC2	-1.61		
